# Primary care in the Calais Jungle

**DOI:** 10.3399/bjgpopen17X100557

**Published:** 2017-01-09

**Authors:** Gerry Clare, Polly Nyiri

**Affiliations:** 1 Consultant ophthalmologist, Western Eye Hospital, London, UK; 2 GP, Health Inclusion Clinic, Guy’s and St Thomas’ Hospital NHS Foundation Trust, London, UK

## Introduction

Last summer our small medical team visited the Calais ‘Jungle’. Since that time much has changed and the camp is being demolished and by the time this article is read, it will probably be long gone. Some youngsters are finally being brought to the UK under the ‘Dubs’ amendment. However, once this camp is cleared it will not solve the ongoing flight of refugees from war torn areas: other camps are already appearing.

## July 2016

A young Afghan man caught his finger on a sharp point while trying to cross a barbed wire fence. The finger was partially degloved. He attended the local hospital, where they placed a few sutures, but now, 2 weeks later, the skin is necrotic and the underlying tissue looks infected. He is in danger of losing his finger.

A middle-aged Sudanese man has been having rigors and is generally unwell. He says it is similar to when he last had malaria.

A young Ukrainian woman complains of lower back pain and urinary frequency.

The paths of these three people may never have crossed; yet here they are, denizens of the Calais Jungle. They turn up to a makeshift primary care ‘clinic’ that we set up in the heart of the unofficial refugee camp one weekend in July 2016.

With only basic medical supplies, we are immediately challenged by what we see. How can we arrange secondary care for the young Afghan in danger of losing his finger? We try to persuade him to return to the original local hospital, but he is reluctant. It was not a good experience for him the first time round.

With the other two patients, it is easier. They can attend the Salam clinic run by a local association during weekdays. Later, we receive word that malaria has been confirmed in our Sudanese patient.

**Figure 1.**
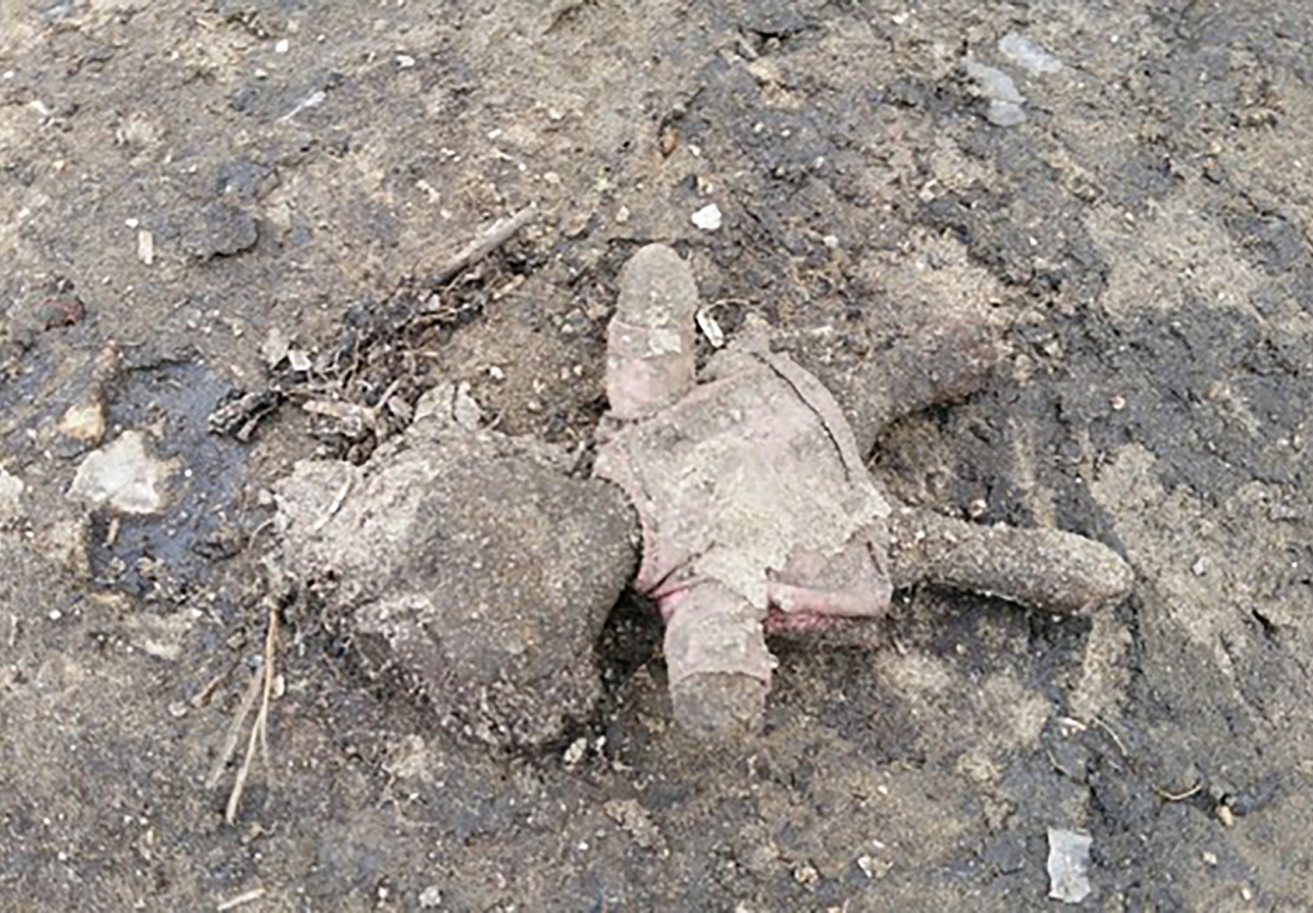

More people arrive, presenting with scabies, rat bites, tinea, chest infections, and wheezing from inhaling smoke from fires lit to cook and keep warm in their tents at night. We examine a severely malnourished 2-year-old boy. We meet several of the camp’s 600 unaccompanied children, at grave risk of sexual exploitation. We learn that there is inadequate safeguarding in place to protect them. A young Eritrean man comes in worried about his eye. He has sustained direct ocular trauma from a rubber bullet, and will never see normally again out of that eye. We see haematomas from police batons, and hear about children being exposed to tear gas again and again (Figure 1).

## The reality

**Figure 2.**
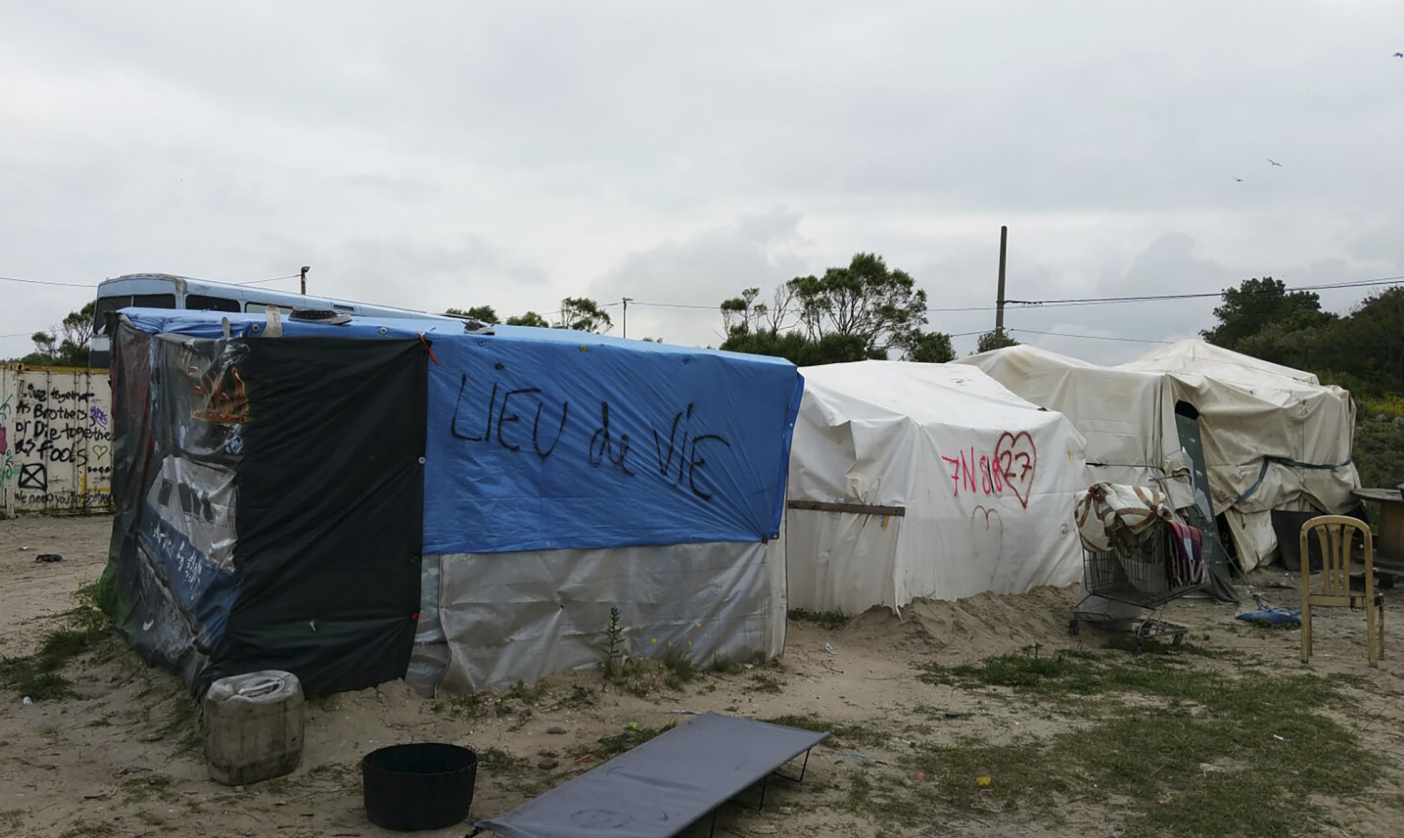

**Figure 3.**
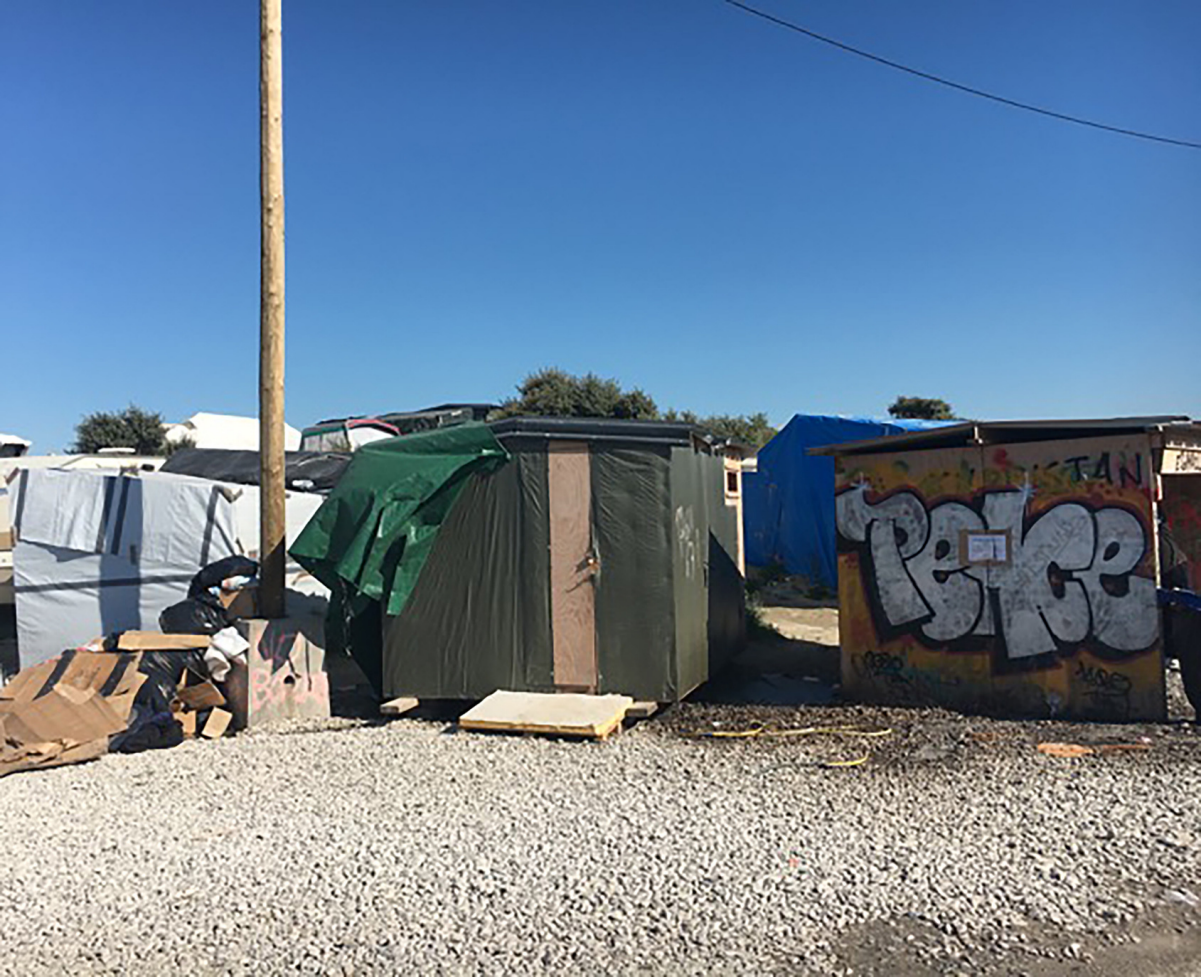

These are no ordinary patients. They have travelled far from home to escape war, poverty, and misery. They have endured personal odysseys to get here, experienced untold hardships, and suffered unimaginable privations. Many have survived the loss of their families, torture, and rape. Their journeys over, for the moment at least, they must make their homes in the Calais Jungle. Their new shelters are in many cases mere tarpaulin covers, and their new beds just rugs on the ground. They own next to nothing. There is little for them to do, besides use their ingenuity to cross the English Channel in search of a better life. They are vulnerable to exploitation, crime, injury, and disease. Potentially violent clashes with local police, with other ethnic groups resident in the Jungle, or local far right agitators who visit at night await them. Set on a former landfill site, the camp is a dustbowl in summer and a freezing quagmire in winter. The stench from the chemical toilets testifies that they are in constant use. Rubbish is strewn everywhere. The charity, Doctors of the World UK, discovered that faecal contamination of the water supplies and outbreaks of diarrhoeal disease are commonplace. This Dantean circle of hell exists on our border in 2016 (Figures 2 and 3).

Our team includes three GPs, an ophthalmologist, and an Arabic interpreter. The GPs normally work in a specialist clinic for refugees in Brixton, London. Our ophthalmologist, a consultant in London, has worked in war zones and as part of *Médecins Sans Frontières*’ response to the Ebola outbreak. Yet we are shocked by the paucity of medical aid, and our contribution, a weekend clinic when other local clinics are closed, feels like a tiny drop in an ocean of want. The lack of a coordinated medical response is striking, and is a scandalous indictment of the apathetic response of our governments to this human tragedy. Thankfully there are others; non-governmental organisations providing the basics to make life for these refugees more bearable.

Alas, the message that is heard in jungles like the one in Calais, where the majority of residents are Muslim, is all too frequently one of rejection and not one of compassion. In today’s world of searing inequality, of disenfranchised and dispossessed youth turning to violence, can we afford to keep transmitting this message? Will it not be counterproductive in the long run?

## The future

**Figure 4.**
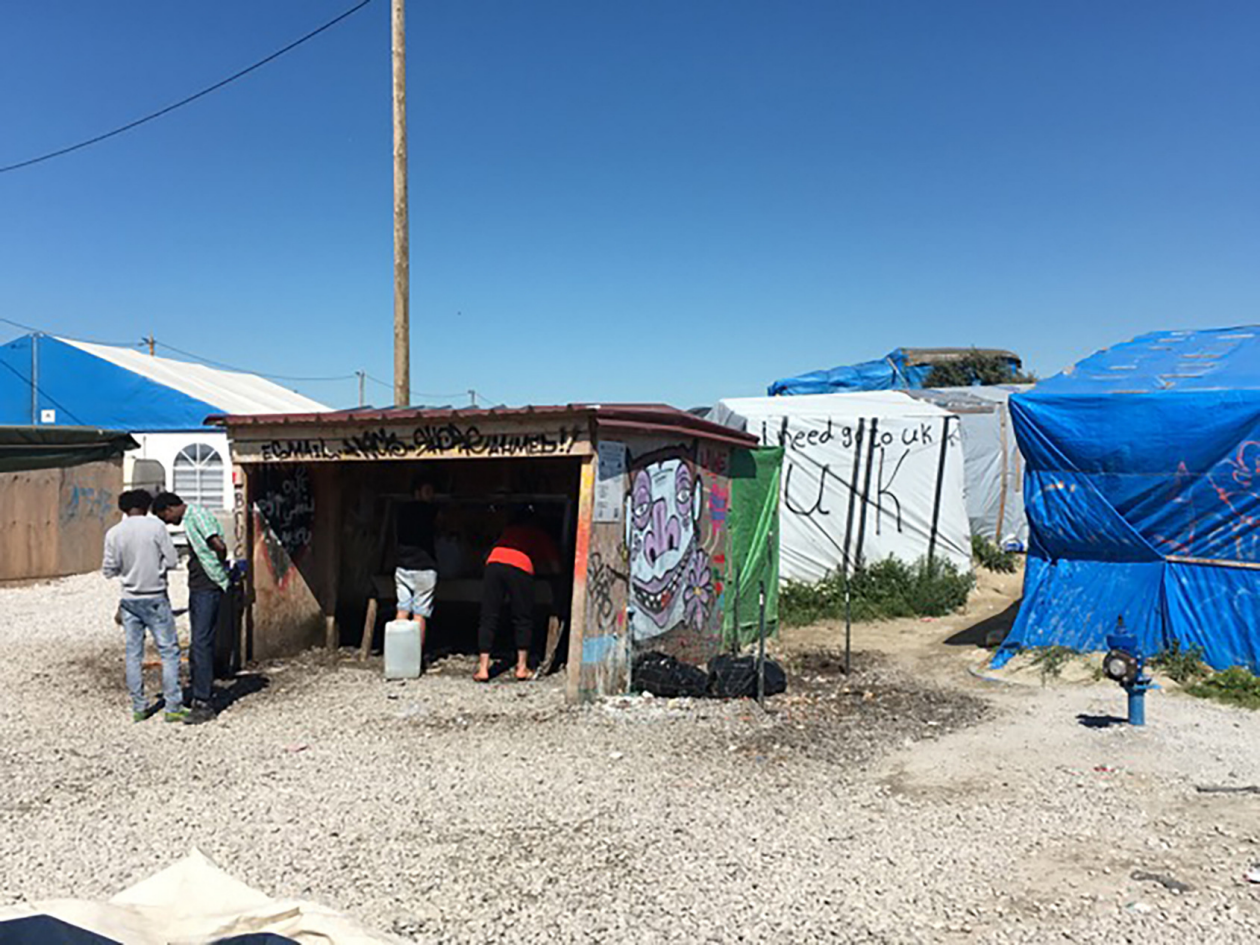

We can conceive of a better response. We would like to see a modern healthcare facility in the Calais Jungle, staffed by volunteers and professionals and managed by government. We call for comprehensive screening of refugees for infectious diseases such as tuberculosis, HIV, schistosomiasis, malaria, hepatitis B, and so on, just as one would expect in the case of entry into the US. Secondary care pathways should be formalised for serious cases, such as that of the young Afghan with the damaged finger. A minimum of medical diagnostic equipment and pharmaceuticals must be provided. One can easily imagine the military, with its abundance of equipment and staff at the ready, providing such a service in collaboration with civilian partners. The UK response to Ebola in Sierra Leone provides a recent example of military assistance to mitigate a public health disaster. Why not here? (Figure 4).

Ideally, we would like to see a concomitant improvement in the social welfare of the residents. We reject the argument that this would provide too much of a ‘pull’ factor for other refugees. People have a right to expect basic humanity, whatever the circumstances. The human right to health cannot simply be marginalised for political expediency.

**Figure 5.**
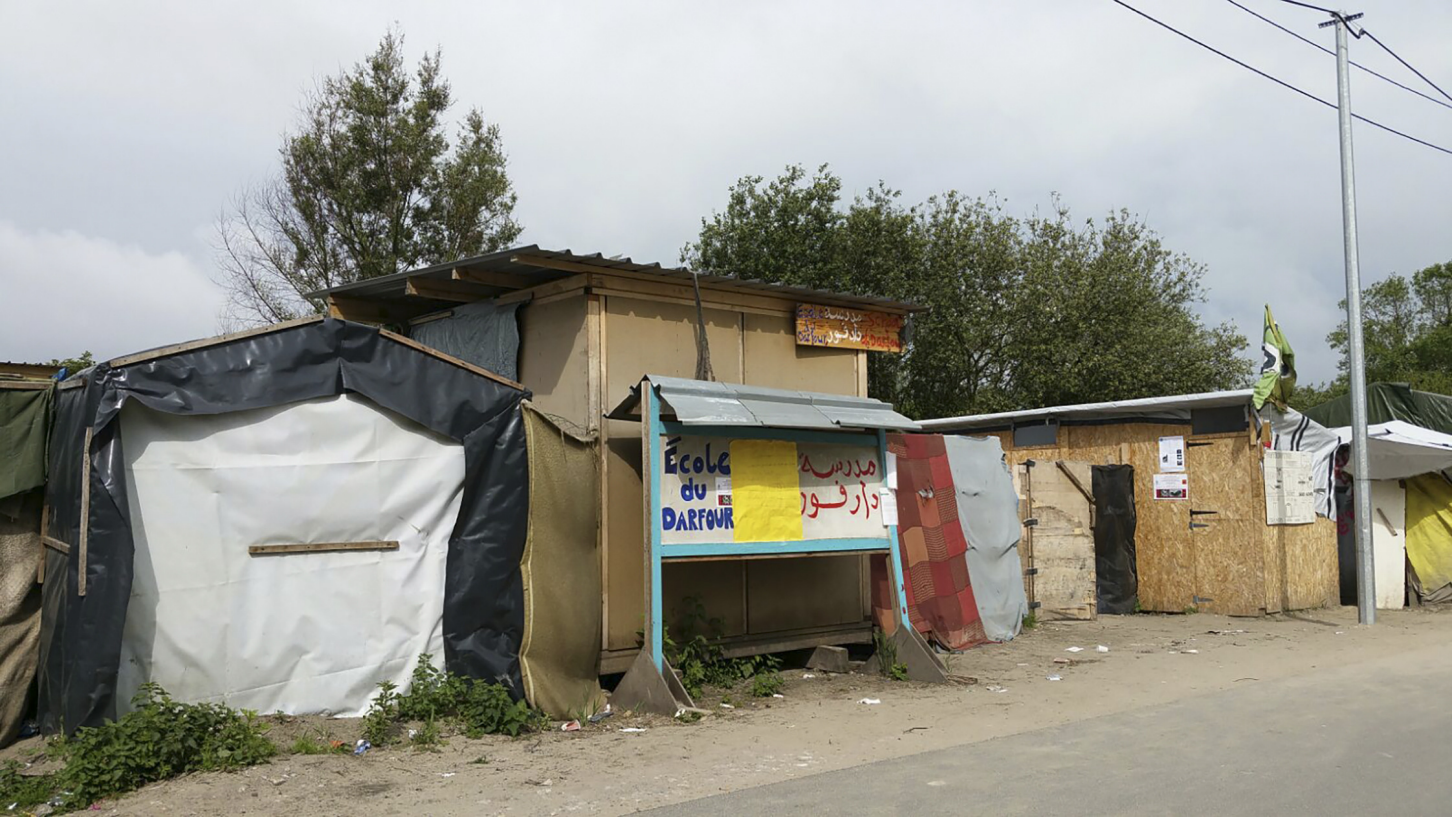

The problems posed by the disadvantaged population in the Calais Jungle are ours to solve, and they are not beyond our wit. We have a right to expect our governments and other stakeholders to work together to achieve an acceptable solution to the plight of these refugees. As doctors, we must stand in unison to demand better conditions for these people, many of whom would be legitimate patients in our surgeries and departments had their journeys been allowed to proceed to completion (Figure 5).

## October 2016

As we write in October, hundreds of riot police are arriving to dismantle the camp and move people on. Refugees are being bussed to reception centres around France. We hope that they are not detention centres. Many are fleeing to join the Dunkirk camp, which is even more squalid than the one in Calais, and to an increasing number of satellite camps that are springing up around the area. The need for medical care will remain just as urgent. Simultaneously, a small number of unaccompanied children are starting to arrive in London, which gives hope for just a few.

A failure to act will not be judged kindly in the fullness of time. Let us not miss this golden opportunity to engage the wider world by pretending that these apocalyptic jungles do not exist. We call for concerted action to provide sustainable medical care for the refugees on our doorstep. Calais will close, but others are reappearing. Volunteer medics are moving to the new camp in Paris.

We call for concerted action to provide sustainable care for the refugees on our doorstep: in Calais and beyond.

